# Noble gas and neuroprotection: From bench to bedside

**DOI:** 10.3389/fphar.2022.1028688

**Published:** 2022-11-29

**Authors:** Haiying Yin, Zijun Chen, Hailin Zhao, Han Huang, Wenwen Liu

**Affiliations:** ^1^ Department of Anesthesiology and Key Laboratory of Birth Defects and Related Diseases of Women and Children, Ministry of Education, West China Second University Hospital, Sichuan University, Chengdu, China; ^2^ Department of Anesthesiology, Southwest Hospital, Third Military Medical University, Chongqing, China; ^3^ Division of Anesthetics, Department of Surgery and Cancer, Pain Medicine and Intensive Care, Faculty of Medicine, Imperial College London, Chelsea and Westminster Hospital, London, United Kingdom; ^4^ Department of Anesthesia Nursing, West China Second University Hospital, Sichuan University/West China School of Nursing, Ministry of Education, Sichuan University and Key Laboratory of Birth Defects and Related Diseases of Women and Children (Sichuan University), Chengdu, China

**Keywords:** xenon, argon, helium, noble gas, neuroprotection, pharmacology

## Abstract

In recent years, inert gases such as helium, argon, and xenon have gained considerable attention for their medical value. Noble gases present an intriguing scientific paradox: although extremely chemically inert, they display a remarkable spectrum of clinically useful biological properties. Despite a relative paucity of knowledge about their mechanisms of action, some noble gases have been used successfully in clinical practice. The neuroprotection elicited by these noble gases has been investigated in experimental animal models of various types of brain injuries, such as traumatic brain injury, stroke, subarachnoid hemorrhage, cerebral ischemic/reperfusion injury, and neurodegenerative diseases. Collectively, these central nervous system injuries are a leading cause of morbidity and mortality every year worldwide. Treatment options are presently limited to thrombolytic drugs and clot removal for ischemic stroke, or therapeutic cooling for other brain injuries before the application of noble gas. Currently, there is increasing interest in noble gases as novel treatments for various brain injuries. In recent years, neuroprotection elicited by particular noble gases, xenon, for example, has been reported under different conditions. In this article, we have reviewed the latest *in vitro* and *in vivo* experimental and clinical studies of the actions of xenon, argon, and helium, and discuss their potential use as neuroprotective agents.

## 1 Introduction

Helium, neon, argon, krypton, xenon, and radon are collectively known as noble gases because they hardly react with any other material at room temperature or at ambient air pressure. However, some noble gases quite “actively” produce various biological effects. As early as the 1950s, xenon was found to produce a general anesthetic effect, both in humans and animals (Cullen and Gross, 1951). Later, it was demonstrated that noble gases can also produce anti-apoptotic effects and protect solid organs from various injuries (Winkler et al., 2016; Anna et al., 2020). Owing to the lack of biological activity of neon (Winkler et al., 2019) and toxic radioactivity of radon (Gray et al., 2009), only helium, argon, and xenon have been evaluated as medical gases for clinical applications, largely as general anesthetics or for organ protection (Oei et al., 2010; Maze, 2016; Gardner and Menon, 2018; Solevag et al., 2019; Wang et al., 2019).

To date, xenon is the best characterized noble gas used in biomedicine. In addition to producing an anesthetic effect, xenon elicited neuroprotective effects against various injuries in both preclinical and clinical models of neonatal hypoxic-ischemic encephalopathy (HIE), spinal cord ischemia, traumatic brain injury (TBI), and even neurodegenerative disorders such as Alzheimer’s disease (AD) and Parkinson’s disease (PD) (Lavaur et al., 2016; Winkler et al., 2016; Le Nogue et al., 2020). At present, the mechanism by which xenon yields neuroprotection is primarily attributed to its competitive inhibition at a glycine site of N-methyl-D-aspartate (NMDA) receptors, although activation of adenosine triphosphate -sensitive potassium (KATP) channels or two-pore potassium channels may also explain some of its neuroprotective effects (Gruss et al., 2004; Bantel et al., 2010). Investigations show that xenon has potential neuroprotective effects at concentrations ranging from 35% to 70% (Yang T. et al., 2012; Zhang et al., 2019). Argon, another noble gas, exhibited neuroprotective effects in organotypic hippocampal cultures isolated from mouse pups subjected to either a purposive focal mechanical trauma or oxygen-glucose deprivation (OGD) (Loetscher et al., 2009). Current evidence suggests that the neuroprotection mediated by argon is linked to its inhibition of Toll-like receptors 2 and 4 (TLR2 and TLR4) or activation of nuclear factor (erythroid-derived 2)-like 2, a mechanism of neuroprotective activity significantly different from that of xenon (Ulbrich et al., 2015a; Zhao et al., 2016a). As for helium, the most striking difference between helium and xenon is that its anesthetic effects can only be achieved at 189 atm—a condition that may account for confounding effects of high-pressure nervous syndrome, making this approach impractical for perioperative application (Dickinson and Franks, 2010). Unexpectedly, various pre- and postconditioning protocols have revealed neuroprotective and cardioprotective effects of helium following ischemia-reperfusion injury (IRI) (Aehling et al., 2018). The aim of this work is to review preclinical (both *in vivo* and *in vitro*) and clinical studies employing helium, argon, and xenon, with a focus on their possible pharmacology, neuroprotection, and clinical applications.

## 2 Xenon

In 1898, xenon was identified as a colorless, tasteless, odorless dense noble gas present in trace amounts in our Earth’s atmosphere, accounting for no more than 0.087 ppm in atmospheric air. Specifically, it was discovered by two chemists, Ramsay and Travers, in the residue left over from evaporated components of liquid air in Britain. Since then, xenon has been developed for many different applications, from gas-discharge lamps and lasers, to medical uses including anesthesia, organic protection, and imaging (Sase et al., 2017).

### 2.1 Pharmacology of xenon

**FIGURE 1:**
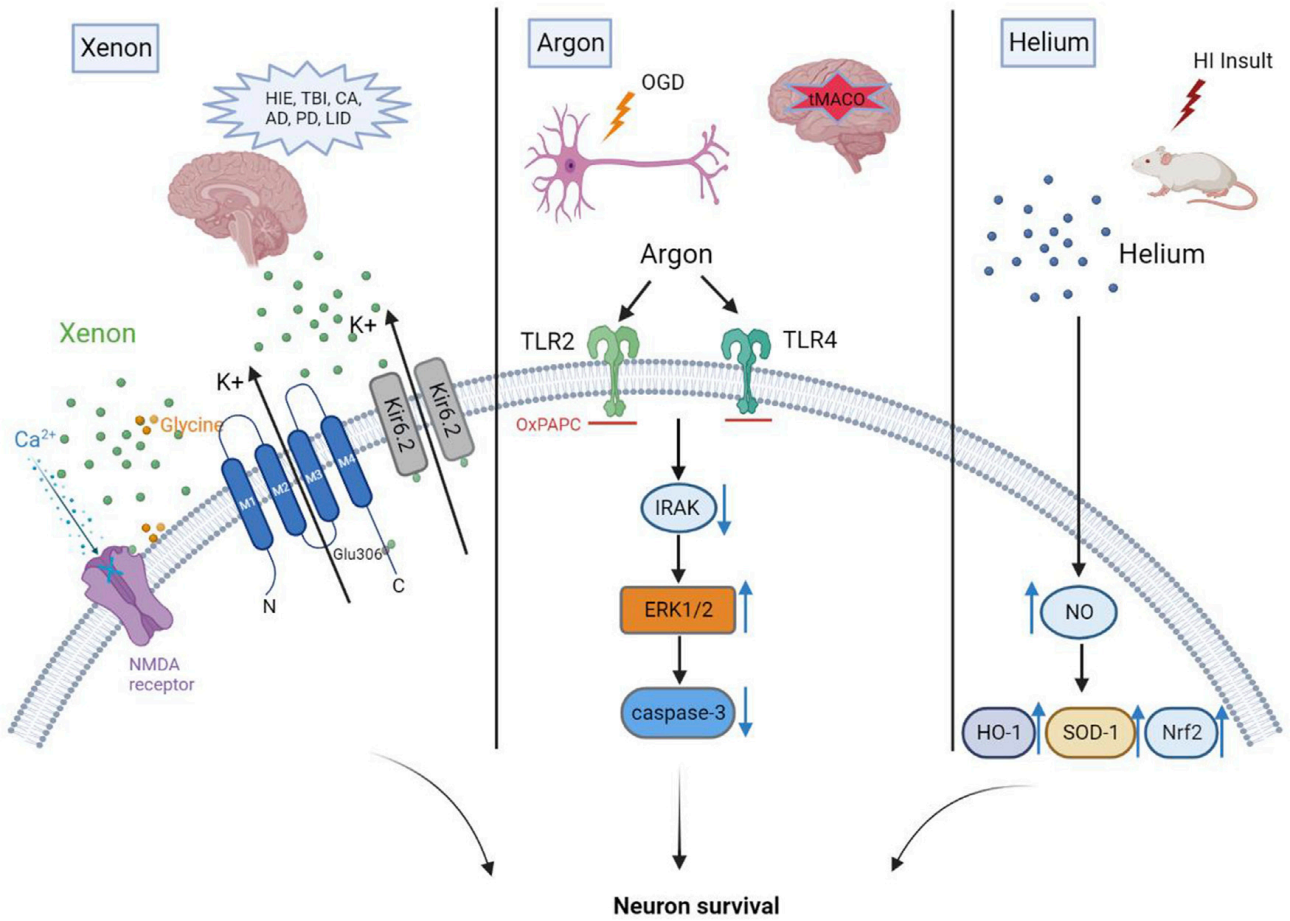
Xenon yields neuroprotection mainly due to its inhibition at a glycine site of NMDA receptors. Neuroprotection mediated by argon is linked to its inhibition of TLR2 and TLR4. Helium preconditioning upregulates the anti-oxidases mediated by nitric oxide (NO) to produce neuroprotective effects.

Unlike other commonly used general anesthetics, such as propofol, benzodiazepines, or inhaled anesthetics, xenon mainly acts on NMDA receptors, with a negligible effect on γ-aminobutyric acid (GABA) (Salmi et al., 2008; Yang et al., 2019). Recent studies identified several molecular targets for xenon that may be responsible for its various attractive pharmacological properties. NMDA receptors, the first identified molecular target, were noncompetitively inhibited by xenon in cultured hippocampal neurons (Franks et al., 1998). Overall, previous study showed that xenon could reduce NMDA-activated currents by 60% at a concentration of 80% (Franks et al., 1998). Binding of xenon at a glycine site of NMDA receptors results in their inhibition, the extent of which was related to the concentration of glycine. The fact that reducing glycine could increase the degree of NMDA receptor inhibition to 80% from below 50% was attributed to the finding that xenon occupies the physiological binding site of glycine in NMDA receptors (Dickinson et al., 2007). TREK-1, a two-pore-domain potassium channel, is a novel target for xenon-induced general anesthesia (Gruss et al., 2004). Mechanismly, evidence suggests that the amino acid Glu306 plays a critical role in the anesthetic effect of xenon, mainly because Glu306 was proven to participate in the modulation of TREK-1 (Gruss et al., 2004). In addition, Xenon acts as a novel KATP channel opener through directly working on the Kir6.2 pore-forming subunit of the channel to reduce ATP inhibition of the channel and enhance KATP currents (Bantel et al., 2010). Xenon is the first KATP channel opener that readily crosses the blood-brain barrier and thus is of great prospect as a potential neuroprotectant. See Figure 1 for xenon neuroprotection against overactivation of NMDA receptor in different brain diseases or injuries.

The safety and efficacy of xenon as a general anesthetic for medical applications in animal and human models has been investigated by numerous researchers from the perspective of NMDA-receptor blockade (Goto et al., 1997; Rossaint et al., 2003; Hofland et al., 2017; Stevanovic et al., 2017; Kulikov et al., 2019). Compared with propofol and volatile inhalation anesthetics, xenon anesthesia rarely produces dramatic fluctuations in blood pressure or significant drops in heart rate (Law et al., 2016; Schäfer et al., 2019). Beyond its properties of rapid induction and emergence from anesthesia, stable hemodynamics and the absence of respiratory or hepatic toxicity make xenon an ideal anesthetic agent (Coburn et al., 2007). However, the rarity of xenon in atmospheric air and extra expense of devices necessary to deliver xenon anesthesia (with which many anesthesiologists are unfamiliar) remain two major obstacles for the promotion of xenon in perioperative clinical use (Law et al., 2016).

Subanesthetic dosages of xenon can actively elicit antinociception. Xenon exerts its analgesic effect mainly through inhibition of NMDA receptors. In animal models, an experiment carried out in rats demonstrated that 79% of xenon-treated rats subjected to formalin injection in the rear paw showed reduced antinociceptive response compared with rats treated with nitrous oxide or oxygen (Fukuda et al., 2002). Moreover, numbers of c-fos-positive cells and phosphorylated NMDA receptor-positive cells in the xenon group were significantly decreased compared with the other groups, validating the hypothesis that the analgesic effect of xenon arises from its inhibition of NMDA receptors (Fukuda et al., 2002). Another animal study suggested that application of low-dose xenon in 0.9% saline could block long-term potentiation, a mechanism through which xenon mediated a preventative effect on the spinal pain pathway (Benrath et al., 2007). In a rat model of intrauterine perinatal asphyxia, researchers investigated both the analgesic and neuroprotective characteristics of xenon (Yang T. et al., 2012). Following preconditions with xenon, intrauterine perinatal asphyxia was alleviated for up to 4 h. Specifically, maternal behavior and spinal cord dorsal horn neuronal activation (measured with c-fos) were evaluated as primary outcomes to appraise the antinociceptive effect induced by a sub-anesthetic dose of xenon. Decreases of both nociceptive behaviors and c-fos expression revealed that low-dose xenon likely acted as an analgesic during labor without related side effects, thus warranting further clinical study. The potential molecular mechanism was attributed to the reduced activity of transient receptor potential vanilloid type 1 (White et al., 2011). As for human studies, two randomized, double-blinded clinical trials adopted a novel and economical method to investigate the antinociceptive properties of xenon at a sub-anesthetic dose (Froeba et al., 2010; Holsträter et al., 2011). Both studies found that either the subjects’ pain tolerance to mechanical, cold, and ischemic stimuli increased after xenon was intranasally administered at a rate of 1.0 L for approximately 30 min, or application of low-dose xenon strikingly reduced intraoperative opioid requirement and postoperative pain. These findings may improve the effectiveness of pain management and provide a new approach for multimodal analgesia to facilitate enhanced recovery after surgery. The apparent cardioprotective and neuroprotective effects of xenon have made it a persistently hot research topic. Overactivation of NMDA receptors is thought to cause ongoing neuronal injury and death in acute processes such as stroke and TBI, as well as during neurodegenerative disorders (Choi et al., 1988). Experiments carried out in animal models of myocardial ischemia reproducibly demonstrate that xenon can reduce the size of myocardial infarction, whether administered before or after myocardial ischemia (Baumert et al., 2007; Schwiebert et al., 2010; Roehl et al., 2013; De Deken et al., 2016). The cardioprotective effects of xenon are related to several molecular targets, including extracellular signal-regulated kinases (ERK) 1/2, p38 mitogen-activated protein kinase (MAPK), MAPK-activated kinase-2, heat-shock protein 27, glycogen synthase kinase 3β, protein kinase B, and protein kinase cƐ (Weber et al., 2005; Weber et al., 2006a; Weber et al., 2006b; Mio et al., 2009). An international, multicenter, Phase III, single-blinded, randomized trial comparing the effect of xenon anesthesia to sevoflurane and total intravenous anesthesia (TIVA) in patients undergoing coronary artery bypass graft surgery found that postoperative cardiac troponin T (cTnT) release was similar in xenon and sevoflurane groups, but significantly lower than the TIVA group. These results suggest that xenon has similar potential to protect against myocardial damage as sevoflurane (Hofland et al., 2017). However, because this study was conducted in low-risk patients, it should be determined whether xenon can decrease levels of postoperative cTnT release in higher risk patients for whom the benefits provided by xenon anesthesia may be greater. Another human study found that inhaling xenon at an end-tidal concentration of 40% combined with hypothermia (33°C) could reduce cTnT release to a large extent compared with hypothermia alone in comatose survivors of out-of-hospital cardiac arrest (CA) (Arola et al., 2017). Additionally, this study showed that the cardioprotective effect of xenon was hardly affected by age, sex, or performance of percutaneous coronary intervention (PCI). In the group treated with combined xenon and hypothermia, there was almost no difference in cTnT release of patients with or without PCI, or with acute coronary syndrome. This study not only demonstrated the cardioprotective effects of xenon, but also proved that xenon is an apparently independent factor attenuating the severity of myocardial ischemia following out-of-hospital CA (Arola et al., 2017). In addition to cardioprotective and neuroprotective effects, xenon was found to protect against lung injury in a rat model of kidney transplantation (Hailin Zhao et al., 2015).

Although xenon has great potential for medical applications, as demonstrated by a series of animal experiments and clinical studies, disadvantages of this approach still exist. For example, xenon antagonizes 5-hydroxytryptamine type 3 receptors, which mediate nausea and vomiting, and thus may cause postoperative nausea and vomiting (PONV) (Suzuki et al., 2002). A post-hoc explorative analysis revealed that xenon anesthesia is closely related to nausea, and even administration of 4 mg dexamethasone during the first hour of xenon anesthesia had little effect on reducing the incidence of PONV after surgery (Fahlenkamp et al., 2016). Statistics from a meta-analysis reanalyzing several studies comparing xenon with other inhaled anesthetics or propofol drew a final conclusion that the incidence of PONV was higher when general anesthesia was conducted with xenon (Law et al., 2016).

### 2.2 Neuroprotection by xenon

#### 2.2.1 Effect of xenon on perioperative neurocognitive disorders

Xenon has the advantage of reducing the incidence of perioperative neurocognitive disorders (PNDs). Notably, GABAergic signaling partially interacts with PND risk factors, as observed for many inhalational anesthetics (Brohan and Goudra, 2017). However, xenon does not affect GABA receptors (Preckel et al., 2006), making it a promising inert gas to minimize PNDs. A study conducted in mice showed that pretreatment with a 70% concentration of xenon for 20 min before administration of 1.8% isoflurane anesthesia largely attenuated memory deficits induced by surgery on the right hindlimb tibia when compared with isoflurane anesthesia alone (Vizcaychipi et al., 2011). This effect of xenon on preventing memory deficits may be explained by a reduction of interleukin β levels in plasma and upregulation of heat shock protein 72 in the hippocampus (Vizcaychipi et al., 2011). In a long-term follow-up study in mice subjected to controlled cortical impact, measurements of cognitive performance by various contextual fear conditioning paradigms showed no significant difference between xenon and sham groups at 20 months after injury (Campos-Pires et al., 2019). Xenon (75%) was administered to mice for 3 h commencing 15 min after brain injury; cognitive functions, especially memory, were greatly improved when tested 20 months later (Campos-Pires et al., 2019). Prolonging the duration of xenon treatment is predicted to yield more beneficial outcomes, but it is difficult to quickly assess whether xenon plays a critical role in preventing PNDs. A clinical study showed that xenon anesthesia both shortened the emergence time from anesthesia and improved postoperative cognitive dysfunction of patients during the early postoperative period compared with sevoflurane (Bronco et al., 2010). Indeed, as fast recovery from xenon anesthesia shortens the total exposure time to general anesthesia, we may intend to take the improvement of PNDs produced by xenon for granted. However, results concerning the effect of xenon anesthesia on PNDs are mixed. Two studies carried out in patients aged 65–75 years (American Society of Anesthesiologists scores I–III) undergoing elective surgery indicated that xenon did not improve PNDs in the elderly, probably resulting from the short follow-up time after surgery (Coburn et al., 2007; Cremer et al., 2011). An international multicenter randomized, double-blind, parallel-group, controlled clinical trial conducted in high-risk patients scheduled for hip fracture surgery provided further observation of PNDs up to 4 days after surgery (Coburn et al., 2018). Bispectral index (BIS) technology was applied to maintain a proper depth of anesthesia in both groups and prevent it from being a confounding factor in study. The incidence of PNDs in xenon-anesthetized patients was 33% lower than sevoflurane-anesthetized patients, but not statistically significant. Although there was no statistically significant difference between xenon and sevoflurane groups, the 33% reduction still manifests a clinical benefit for elderly patients who are at high risk of developing PNDs undergoing perioperative hip fracture surgery. The study fails to suggest that xenon anesthesia improved the incidence of PNDs compared with sevoflurane anesthesia, which may be attributed to the relatively strict inclusion criteria and use of BIS monitoring (Coburn et al., 2018). Layth Al et al. conducted a randomized, observer-blind, controlled clinical trial to investigate whether xenon anesthesia could reduce the incidence of PNDs in older patients who were screened daily for 5 d after on-pump cardiac surgery (Al Tmimi et al., 2020). Because 40%–60% xenon anesthesia was administered just before and after cardiopulmonary bypass, which is a relatively short period that takes up only one-fifth of the whole anesthesia time, this study did not detect an advantage of xenon anesthesia over sevoflurane anesthesia for reducing the incidence of PNDs. Moreover, use of propofol during cardiopulmonary bypass may negatively influence the effects of xenon because propofol has been proven to provoke neuro-apoptosis in neonatal mouse brain (Tagawa et al., 2014). Similarly, there is no exception for pediatric populations. A Phase II mono-center, prospective, single-blind randomized controlled pilot trial carried out in children aged 4–12 years suffering from congenital heart disease and undergoing heart catheterization showed remarkable neurocognitive impairments shortly after anesthesia. Administration of xenon as an adjuvant to sevoflurane anesthesia did not reverse this effect to a larger extent than sevoflurane anesthesia alone (Devroe et al., 2018). Therefore, further preclinical studies and clinical studies targeting certain diseases are merited to explore potential actions of xenon on cognitive function.

#### 2.2.2 Xenon elicited neuroprotection in neonatal hypoxic ischemic encephalopathy

In recent years, research interest was renewed by the discovery that xenon may act as a neuroprotective agent by antagonizing NMDA receptors. Overactivation of NMDA receptors leads to an unpredictable amount of calcium influx that triggers cellular cascades and, ultimately, kills brain cells. Xenon has been proven to exert neuroprotective properties in both *in vivo* and *in vitro* models, including HIE in neonates, TBI, stroke, cerebral ischemia following CA, AD, PD, and L-dopa-induced dyskinesia (LID) (Thoresen et al., 2009; Chakkarapani et al., 2010; Arola et al., 2013; Campos-Pires, 2013; Campos-Pires et al., 2015; Azzopardi et al., 2016; Laitio et al., 2016; Lavaur et al., 2016; Campos-Pires et al., 2018; Campos-Pires et al., 2019; Le Nogue et al., 2020). The technique of applying moderate hypothermia (33°C–34°C) is widely adopted for the treatment of neonatal asphyxia, becoming a standard strategy in some countries (Azzopardi et al., 2012). However, roughly a quarter of infants treated with hypothermia die and one in five survivors develop irreversible central nervous system damage, including sensorimotor and cognitive impairments. To overcome with this problem, multiple therapies have been explored, among which xenon stands out in a comparative review (Robertson et al., 2012). Xenon provides neuroprotection against hypoxia-ischemia (HI) by competitively inhibiting NMDA receptors at a specific glycine site (Banks et al., 2010). Caspase-3 immunostaining showed that xenon remarkably reduced neuronal apoptosis by activating anti-apoptotic factors and prevented isoflurane-induced apoptosis in a dose-dependent manner (Ma et al., 2007). In a healthy newborn pig model, neuro-apoptosis was not observed following exposure to 50% xenon for 24 h, a preclinical result that supports clinical trials for xenon neuroprotection in neonates (Sabir et al., 2013). John and colleagues carried out two studies in rats and pigs using a combination therapy of 50% xenon and hypothermia. Their results showed that neuroprotection doubled from 35% to 70% when xenon was added to hypothermia immediately after HI insult (Hobbs et al., 2008; Chakkarapani et al., 2010). However, it remains difficult in clinical practice to simultaneously implement initiation of cooling and xenon therapies shortly after HI insult. Thus, xenon preconditioning or postconditioning with a delay of several hours may be more in line with clinical settings. Xenon preconditioning, which refers to administration of xenon before HI insult, has been proven to exert neuroprotective effects as evidenced by improvements of histology and neurologic function in both *in vitro* and *in vivo* models (Ma et al., 2006). The neuroprotective effects of xenon were also investigated in a study of 7-day-old Wistar rat pups undergoing a unilateral HI insult (Liu et al., 2015). The Wistar rat pups received 5 h of xenon treatment following 5 h of hypothermia after a delay of 5 h from HI insult. The results revealed that combining 5-h delayed xenon with delayed hypothermia treatment improved long-term motor and cognitive functions (Liu et al., 2015). These findings extend the therapeutic window, giving doctors more time to diagnose and intervene neonatal HIE.

Recently, a feasibility study found that infants with encephalopathy given 50% inhaled xenon for 18 h combined with 72-h therapeutic hypothermia showed no adverse mental or physical effects during an 18-month follow up (Dingley et al., 2014). However, a proof-of-concept, open-label, randomized controlled trial of 92 infants conducted in four intensive-care neonatal units in the United Kingdom compared the neuroprotective effect of moderate hypothermia plus inhaled xenon with moderate hypothermia alone after birth asphyxia within 6 h of birth (Azzopardi et al., 2016). To evaluate the neuroprotection elicited by xenon, the ratio of cerebral lactate to N-acetyl aspartate in the thalamus was estimated by magnetic resonance spectroscopy and fractional anisotropy (a measure of tissue integrity) of white matter tracts was measured using diffusion tensor magnetic resonance imaging. Quantification of brain-damage biomarkers was not significantly altered; therefore, no treatment benefit was manifested. Perhaps eligible newborns were in a state of severe asphyxia and hence there is little expectation of benefit from any medical intervention. Another explanation is that the elected biomarkers were not sensitive enough to detect any benefits.

#### 2.2.3 Xenon provides neuroprotection following cardiac arrest

Preclinical studies showed that xenon can provide neuroprotection in models of CA. In the first study, CA induced with an alternating current resulted in ventricular fibrillation in pigs. Following 8-min of untreated CA, 5-min precordial compression, and defibrillation, pigs were considered to be successfully resuscitated on the condition that mean aortic pressure was higher than 60 mmHg for at least 5 min. One hour later, resuscitated animals were randomly divided into three groups: one received 70% xenon and 30% oxygen ventilation for 1 h (1-h Xe group), one received 70% xenon and 30% oxygen ventilation for 5 h (5-h Xe group), and one received 70% nitrogen and 30% oxygen ventilation for 5 h (control group) (Fries et al., 2008). Compared with the control group, neurologic deficit scores (used to estimate neurologic function) were lower in the 1-h Xe group, and more significantly reduced in the 5-h Xe group at 1–3 days after CA. Notably, there were apparent trends toward decreased neural injuries in the hippocampal and striatal areas in both xenon-treated groups. Although xenon administration yielded promising histopathological findings, improvements of neurological function in xenon groups were transient (benefits were only observed during the first 3 d following resuscitation). Subsequent research revealed that the interval between resuscitation and initiation of xenon treatment was a determining factor for the efficacy of xenon (Derwall et al., 2008; Fries et al., 2009). Delaying xenon application for 1 h ameliorated neurological deterioration, while early xenon application (10 min after resuscitation) did not bring about any functional neurological benefit. Xenon is proven to augment the effect of hypothermia on neurological outcomes. In a study by Fries et al., ten pigs subjected to 6-min CA followed by 10-min cardiopulmonary resuscitation (CPR) were randomly allocated to receive 16-h treatment with mild therapeutic hypothermia (MTH) with or without 1 h of 70% xenon ventilation or normothermia (Fries et al., 2012). Neurologic deficit scoring was employed to evaluate neurocognitive performance for 5 d after CPR, and then pigs were sacrificed to harvest their brains for histopathological analysis. Although necrotic lesions in the hippocampus and putamen were significantly reduced in both treatment groups, combination of MTH with 70% xenon greatly reduced astrogliosis, microgliosis, and perivascular inflammation in the putamen. Moreover, pigs treated with both MTH and xenon exhibited much better neurologic outcomes compared with the other two groups 3–5 d after resuscitation. Hence, the study concluded that adding xenon to MTH improves neurological function and recovery (Fries et al., 2012). In 2016, a human study of 110 comatose patients surviving an out-of-hospital CA (designed by the Xe-HYPOTHECA Study Group) compared neuroprotection produced by xenon in combination with hypothermia (33°C) with that produced by hypothermia alone (Laitio et al., 2016). Patients underwent ventricular fibrillation or non-perfusing ventricular tachycardia and then returned to spontaneous circulation within ≤45 min; the xenon group received an end-tidal xenon concentration of 40% for 24 h shortly after randomization. The results of this clinical trial showed less white matter damage (as assessed by fractional anisotropy of diffusion tensor magnetic resonance imaging) in comatose survivors treated with xenon and hypothermia. However, statistics for neurological functions and mortality at 6 months displayed no significant differences. The Xe-HYPOTHECA Study Group also measured cTnT levels in the same study population at hospital admission and 24, 48, and 72 h post-CA, which revealed that a combination of inhaled xenon with hypothermia reduced myocardial injury and was unaffected by whether patients underwent PCI.

#### 2.2.4 Xenon elicited neuroprotection against traumatic brain injury

Xenon also has a protective effect on TBI. Campos-Pires et al. investigated the effect of xenon on neurological function in a mouse model of TBI (Campos-Pires et al., 2015). Using xenon dosages ranging from 30% to 75%, they found that 30% xenon was effective for the treatment of TBI, making it flexible to increases of oxygen concentrations when needed. Notably, xenon concentrations as high as 70% administered 15 min after TBI could reportedly improve relative long-term neurologic functions measured at 1 month (Campos-Pires et al., 2015). Campos-Pires et al. performed a 20-month follow-up study to observe the effects of 75% xenon on long-term outcomes and survival (Campos-Pires et al., 2019). White matter loss in the contralateral corpus callosum and neuronal loss in the contralateral hippocampal CA1 and dentate gyrus areas were decreased 20 months after treatment with 75% xenon (Campos-Pires et al., 2019). A previous study by Campos-Pires et al. suggested that xenon had neuroprotective characteristics in an organotypic brain slice culture model of TBI, likely mediated through the inhibition of NMDA receptors (Harris et al., 2013). Another *in vitro* study from the same team using a novel blast TBI model for the first time drew the same conclusion (Campos-Pires et al., 2018). There was no apparent difference between 50% xenon-treated mouse organotypic hippocampal brain-slice cultures and sham slices at 24 h or 72 h after blast exposure. This indicates that xenon may reduce the primary injury and prevent subsequent injury development *in vitro*, thus reinforcing the idea that xenon may have an irreplaceable role in treatment of patients with blast-induced TBI. Xenon has also been shown to protect the spinal cord from IRI, which frequently occurs after ascending aortic aneurysm surgery or aortic dissection, leading to acute or delayed paraplegia (Yang Y. W. et al., 2012; Yang et al., 2014). The findings of these two studies suggest that administration of xenon immediately or 2 h after reperfusion provides a neuroprotective effect to the spinal cords of IRI model rats, with the most significant effect observed 1 h after xenon administration (Yang Y. W. et al., 2012; Yang et al., 2014).

#### 2.2.5 Xenon provides neuroprotection against neurodegenerative diseases

In the field of neurodegeneration, a review concluded that excessive activation of extra-synaptic NMDA receptors may be associated with the development of AD and Huntington’s disease, while activation of tNMDA receptors inside synapses increases the possibility of cell survival (Parsons and Raymond, 2014). Should any alteration in this balance enhance synaptic or reduce extra-synaptic NMDA signaling, chances are that the deterioration of these neurodegenerative diseases will be altered (Parsons and Raymond, 2014). Experiments to explore the neuroprotective properties of xenon in two model systems (cortical cultures and cholinergic cultures) mimicking degenerative changes linked to AD pathology demonstrated for the first time that xenon can provide partial but sustained protection of cortical and cholinergic neurons vulnerable in AD. Moreover, xenon exerted cooperative neuroprotection with two noncompetitive NMDA receptor channel antagonists, memantine and ketamine (Lavaur et al., 2016). A glimpse of the neuroprotection provided by xenon against PD has also been observed. Levodopa is generally the first choice for neurologists to treat patients with PD. Unfortunately, LID frequently occurs with administration of this drug. A study conducted in several species to identify the relationship between xenon and PD, as well as LID, elucidated for the first time that xenon can reverse maladaptive plasticity of corticostriatal glutamatergic projections associated with LID (Baufreton et al., 2018). Dyskinesia in rat and nonhuman primate models of PD, as well as gait performance in a nonhuman primate model of PD, were also improved in the same study. Progressive loss of dopamine (DA) neurons is an important mechanism in the pathogenesis of PD. An *in vitro* study using midbrain cultures found that xenon was a robustly neuroprotective agent for DA neurons (Lavaur et al., 2017). The protective effect of xenon for DA neurons was concentration-dependent, as evidenced by optimal effects at concentrations ranging from 50% to 75% (Le Nogue et al., 2020). These findings pave the way for future clinical studies and shine a light on applications of xenon for AD, PD, and LID. However, there may be a long road to applying xenon for PD because it is difficult to determine the proper timing to initiate xenon treatment during the development of neurodegenerative diseases in humans.

### 2.3 Practicable applications of xenon in experimental or clinical settings

John et al. developed an automated gas controller to monitor changes of breathing gas composition recirculated in a closed-circuit system and inject oxygen, air, or xenon as required to make the gas mixture achieve preset values (Dingley et al., 2020). The automated controller successfully maintained the concentration of inhaled xenon close to 50% for 72–78 h at a xenon cost of $11.1/h in a model of hypoxic neonatal piglets (Dingley et al., 2020). A previous study by John et al. described a single-use closed-circuit Xe delivery system that proved both feasible and cost-efficient (Chakkarapani et al., 2009). This closed-circuit xenon delivery system provided access for newborn pigs subjected to 45-min global hypoxic insult to receive 50% xenon treatment for up to 16 h at a cost of < $2/h (Chakkarapani et al., 2009). This research team also invented a portable xenon delivery apparatus, with the aim of allowing xenon inhalation and therapeutic hypothermia as early as possible during ambulance travel to the hospital or transfer from a ward to intensive care unit (Dingley et al., 2015). Apart from the closed-circuit xenon delivery system, researchers developed a new method to delivery xenon to patients. In a study of out-of-hospital CA survivors, researchers tried to deliver xenon to patients in the form of xenon-containing echogenic liposomes (Xe-ELIP) and investigated the time window and optimal dosage of Xe-ELIP therapy (Peng et al., 2013). It concluded that the neuroprotection elicited by Xe-ELIP lasted for 5 h after stroke onset, with the most effective dosages of 7–10 mg/kg (Peng et al., 2013).

## 3 Argon

Argon, located in the eighth group of the third cycle in the periodic table of elements, is a noble gas with an atomic number of 18. A colorless, odorless, and particularly inert gas, argon plays inactive roles in chemical reactions. Importantly, the amount of argon ranks first among the noble gases, with approximately 9,340 ppm in the atmosphere (Ballentine, 2007). Although classified as an inert gas, argon is biologically active, as evidenced by interactions with large proteins and receptors (Růzicka et al., 2007).

### 3.1 Pharmacology of argon

As a non-polar molecule, argon can easily diffuse into tissues, such as deep brain compartments, whereby it can interact with mostly amphiphilic proteins (even within receptor cavities) to induce biological actions such anesthesia and organ- or neuroprotective effects. The potential mechanism by which argon enacts such effects has mainly been explored *in vitro* studies and further confirmed in animal models. Mouse cortices, hippocampal tissues, or coronal tissues subjected to OGD are frequently employed to investigate the biological properties of argon *in vitro*. OGD-related injury result in neural death through oxidative stress (Lu et al., 2011). The nuclear factor erythroid 2-related factor 2 (Nrf2) is an important regulator of cellular resistance to oxidants (Ma, 2013). Studies showed that activation of ERK1/2 pathway could enhance the production of Nrf2 through p-mTOR. In the model of OGD, researchers observed increased expression and translocation of Nrf2 into nuclei after argon exposure, which implies the activation of antioxidant system (Zhao et al., 2016a). Zhao et al. also find that argon treatment markedly reduced production of reactive oxygen species after OGD challenge through flow cytometry, and consequently, the expression of cleaved caspase-3 was decreased (Zhao et al., 2016a). Argon treatment enhanced expression of antioxidant enzymes. Immunofluorescence analysis demonstrated that p-mTOR, Nrf2 and its downstream effectors including NAD(P)H dehydrogenase (quinone 1) (NQO1) and superoxide dismutase 1(SOD-1) protein expression levels were also increased considerably in hypoxic-ischemia rat brain cortex after argon treatment (Zhao et al., 2016a). What’s more, argon significantly upregulate the heme oxygenase-1 (HO-1), a protein protecting neurons against oxidative injury (Chen et al., 2000), in both the cortex and hippocampus of the neonatal hypoxia–ischemia brain (Zhao et al., 2016b). *In vivo*, transient middle cerebral artery occlusion (tMACO) models are frequently applied in rodents. In 2003, Abraini et al. analyzed the effect of argon on related receptors to explore, for the first time, the underlying mechanism of argon. Their results demonstrated that argon exerts its protective effect through the GABAA receptor (Abraini et al., 2003). A study exploring the neuroprotective effect of argon on hippocampal brain slices in a model of traumatic injury offered evidence that neither NMDA or TREK-1 receptors are related to argon-induced neuroprotection (Harris et al., 2013). To analyze whether mitochondrial KATP-channels are responsible for the neuroprotective effect of argon, Brücken et al. administered a 9-mg/kg bolus of 5-hydroxydecaonate to block KATP-channels 50 min after the return of spontaneous circulation and 10 min before starting 70% argon ventilation in a model of CA (Brücken et al., 2014). Although strong neuroprotection was observed, they concluded that 5-hydroxydecaonate (a KATP channel inhibitor) did not influence the effect of argon, indicating that argon did not open KATP channels.

Later attempts to identify receptors associated with the neuroprotection of argon employed neuroblastoma cells *in vitro* and IRI of rat retina *in vivo*. Ulbrich et al. added rotenone to a neuroblastoma cell line to inhibit the mitochondrial respiratory chain and, thus, induce apoptosis (Ulbrich et al., 2015a). Following application of argon concentrations ranging from 25% to 75% to these cells, they found that xenon mediated significant neuroprotection with an optimal effect at 75%. In this *in vitro* study, immunohistology confirmed the results of flow cytometry suggesting that surface expression of TLR2 and TLR4 were largely reduced by argon (Ulbrich et al., 2015a). In other words, the neuroprotection elicited by argon was completely attenuated by inhibition of TLR2 and TLR4. One year later, Ulbrich et al. published their *in vivo* work suggesting that 75% argon inhaled for 2 h protected retinal ganglion cells and decreased retinal TLR2 and TLR4 expression in a model of retinal IRI (Ulbrich et al., 2016). Thus, argon modulated molecular pathways involved in cell survival to exert anti-apoptotic properties. Studies investigating mechanisms of argon-mediated neuroprotection subsequently emerged. In short, argon increases ERK1/2 phosphorylation, blocks the apoptosis cascade (Fahlenkamp et al., 2012; Ulbrich et al., 2015b; Zhao et al., 2016a), and upregulates expression of B-cell lymphoma-2 (Bcl-2, an anti-apoptotic protein) (Zhao et al., 2016b). Argon also activates TLR2 and TLR4 to reduce caspase-3 activity (Zhao et al., 2016b; Ulbrich et al., 2016) and mediate intracellular signaling associated with elevation of pro-inflammatory cytokines, growth factors, and cell survival factors (Ulbrich and Goebel, 2016). Among these preclinical studies, ERK1/2 phosphorylation appears to have a crucial role in the effect of argon because it mediates anti-apoptotic signaling through regulation of BAX, Bcl-2, and caspase-3 activity.

### 3.2 Argon neuroprotection

*In vitro* studies demonstrated that argon has a dose-dependent protective effect on the central neuronal system, both in models of OGD and TBI, with the most beneficial effect at a concentration of 50% argon (Loetscher et al., 2009). Yu-Mi Ryang et al. designed a controlled laboratory study to investigate the neuroprotection provided by 50% argon in male adult Sprague-Dawley rats (Ryang et al., 2011). Their results provided the first evidence that argon exerts a neuroprotective effect in an *in vivo* model of acute focal cerebral ischemia. Two hours of transient tMCAO a well-established and less invasive endovascular technique) was applied to 22 rats to establish an intraluminal filament occlusion model. To make the laboratory experiment more in line with a clinical emergency scenario, argon was administered 1 h after tMCAO. Rats that spontaneously breathed 50% argon through a face mask for 1 h showed significant reductions of both cortical and subcortical infarct volumes, as well as adverse outcomes evaluated 24 h after reperfusion. Because the time of argon treatment and observation window were not long enough in the study conducted by Yu-Mi Ryang and colleagues, another study analyzed the extent to which argon affords neuroprotection and improves neurologic outcomes after 24-h administration in a model of permanent or temporary focal cerebral ischemia established by MCAO (Ma et al., 2019). Three experimental designs were employed, including two models of severe and moderate stroke *via* permanent MCAO without reperfusion and one model of focal ischemia (tMCAO) with reperfusion for 1.5 h. In these three experiments, 70% argon was administered to rats with severe stroke and focal ischemia immediately after stroke induction surgery, but administered to rats with moderate stroke with a delay of 2 h. Among the three main primary outcomes, neurological outcomes and overall recovery were apparently improved by argon in all experiments, whereas infarct volumes were only significantly reduced by argon in the severe stroke model. Improved neurological outcomes and the absence of a reduction in infarct size in the model of focal ischemia (tMCAO) with reperfusion differed from the results of a similar study carried out by Ryang et al. (2011). Differences in administration times (24 h vs 1 h) and concentrations (70% vs 50%) of argon, as well as observation times (7 d vs 24 h) may account for these discrepancies. In another study of 3-h tMCAO induction and 1-h reperfusion, 21 male Wistar rats received either 50%/50% argon/O2 or 50%/50% N2/O2 for 1 h (Liu et al., 2019). Neurologic deficits assessed 7 d after stroke were greatly alleviated and neurons at the ischemic boundary zone were preserved. Further results demonstrated that argon promoted a switch of microglia/macrophage polarization towards the M2 phenotype, which plays a vital role in brain recovery processes including neurogenesis, axonal remodeling, angiogenesis, oligodendrogenesis, and remyelination (Hu et al., 2015).

Argon has the potential to reduce areas of cortical and subcortical damage in animals subjected to global ischemia in a model of CA. In a study of Sprague–Dawley rats, Brücken and colleagues found that 1 h of 70% argon initiated 1 h after 7-min CA and 3-min CPR reduced histopathological damage to the neocortex and hippocampal CA3/CA4 regions, and improved neurological deficit scores compared with the control group during the 7-d follow up (Brücken et al., 2013). One year later, they concluded from another study using the same model of CA that argon provided neuroprotection when treatment was delayed for 3 h (Brücken et al., 2015).

Argon has also been observed to elicit neuroprotection in porcine after CPR (Ristagno et al., 2014). Pigs that underwent 8-min ventricular fibrillation without treatment followed by 5 min of CPR and 70% argon for 4 h showed fast and complete recovery in the subsequent 3 d, as evidenced by neurologic deficit and alertness scores. However, the improvements induced by argon treatment were mainly seen in functional recovery, while there were nearly no histopathological alterations. The relatively short interval between argon inhalation and histopathological evaluation may account for this result. Another reason could have been the selection of brain regions to assess histopathological changes. Although the hippocampal CA1 sector is well recognized to have a close relationship with post-resuscitation cognitive dysfunction in humans, brain damage was more detectable in the putamen or caudate nucleus. In contrast to xenon, argon combined with MTH did not produce a neuroprotective effect in a rat model of CA. In this study, 21 male rats were allocated randomly to receive 6 h of MTH, MTH plus 1 h of 70% argon, or no treatment following 9-min CA and 3 min of CPR (Brücken et al., 2017). The combination of argon and MTH did not improve functional recovery after CA in rats and may have worsened neurologic function (Brücken et al., 2017). This contradicts the finding that inhalation of 45%–50% argon for 2–26 h augmented hypothermic protection 48 h after HI in a neonatal piglet model. This model, which resembles the setting of HIE, is contrary to the scenario of global IRI after prolonged CA and thus may present a distinct result.

In an *in vitro* model of brain trauma, argon reportedly displayed neuroprotective effects when applied immediately or 2–3 h after trauma. Loetscher et al. found that 25%, 50%, and 74% argon significantly reduced cell damage in vitro models of TBI or OGD for 72 h after trauma induction (Loetscher et al., 2009). Harris et al. found that argon elicited neuroprotection at a concentration of 50% in a similar TBI model (Harris et al., 2013). Moreover, 50% argon prevented the advancement of secondary injury at 24, 48, and 72 h. Specifically, injury of mouse hippocampal slices following mechanical trauma developed to 32% ± 4% of the total injury by 1 h and 51% ± 8% by 6 h (total injury present at 72 h). This finding stresses that the timing of argon treatment immediately following a trauma plays an important role in the development of secondary injury. Namely, argon can attenuate the emergence of secondary injury after TBI within a 2-h period (Grüßer et al., 2017).

The neuroprotection provided by argon has been extended to retinal ganglion cells subjected to IRI in rats (Ulbrich et al., 2014). Briefly, retinal IRI was initiated in the left eyes of rats by cannulation with a 30-gauge needle to elevate the intraocular pressure to 120 mmHg for 1 h. Subsequently, 1 h of 25%, 50%, or 75% argon (O2 21%, remainder N2) was administered to rats after release of pressure and reperfusion either immediately following retinal IRI or with a delay of 1.5 or 3 h. Retinal tissues harvested from rats 7 d later were subjected to immunohistochemical staining, western blot analysis, and real-PCR, which showed declines in mRNA expression of Bax, Bcl-2, nuclear factor κB (NF-κB), and retinal caspase-3, as well as caspase-3 protein cleavage and especially NF-kB protein expression. Ventilation with 75% argon immediately after IRI was shown to completely diminish ischemic injury, indicating time- and dose-dependent protection of retinal ganglion cells by argon (Ulbrich et al., 2014). Argon was also recently demonstrated to improve the viability and quantity of vital hippocampal neurons in rats following experimental subarachnoid hemorrhage (Höllig et al., 2016). Furthermore, in human studies, data collected from male humans revealed that cerebral circulation and metabolism are unaffected by argon (Grüne et al., 2017), thus providing a basis for clinical trials of argon in the future.

## 4 Helium

Helium heads the noble gases in the periodic table (Weber et al., 2015). The relatively high viscosity of helium accompanied by a density less than oxygen makes it a candidate for reducing respiratory distress and improving respiratory efficiency in patients with airway obstruction, according to the Renault formula (Bigham et al., 2009).

### 4.1 Pharmacology of helium

During the last decade, an array of studies demonstrated that inhalation of helium or heliox (a mixture of helium and oxygen) can be used in many clinical settings such as bronchiolitis, acute asthma exacerbation, and chronic obstructive pulmonary disease. Because helium has high thermal conductivity, heat loss from the body may occur in a room filled with helium. Therefore, metabolism may be reduced because of low body temperature, leading to reduced energy expenditure (Singer, 2007). Compared with xenon and other volatile anesthetics, administration of helium is safer and easier (Uchino et al., 2012). Moreover, helium is devoid of an anesthetic effect and proven to possess a stable hemodynamic profile (Gainnier and Forel, 2006). Findings from numerous experimental studies suggest that helium protects a variety of organs including brain, heart, liver, and kidney from tissue injury (Rizvi et al., 2010; Zhang et al., 2014). The underlying mechanism of neuroprotection elicited by helium was elucidated by Yi Li et al., who observed improvement of the brain neurovascular niche and upregulation of anti-oxidases mediated by nitric oxide (NO) with helium preconditioning (He-PC) (Li et al., 2016a; Li et al., 2016b). In the neonatal hypoxia ischemia rat model, the NO content in the brain was significantly increased after He-PC, revealing that He-PC promoted the production of NO After treating with NO inhibitor before He-PC, the neuroprotection of helium was compromised, which indicated that NO played an essential role in the neuroprotective effect of He-PC (Li et al., 2016a; Li et al., 2016b). NO was found to activate Nrf2. Meanwhile, the protein expression of hemeoxygenase-1 and superoxide dismutase-1, two important downstream anti-oxidases of Nrf2 were increased markedly (Li et al., 2016a).

### 4.2 Helium neuroprotection

The protective effect of helium depends on its concentration and the model employed. Postconditioning with 70% helium significantly reduced brain infarction when administered 2 h after MCAO (Pan et al., 2007). In contrast, delayed postconditioning with 40% helium had no effect. Helium protected the brain against mild ischemia, but not severe ischemia (Zhuang et al., 2012). Rats subjected to 8-min CA did not exhibit improvements in histological or clinical outcomes with administration of 50% helium for 24 h (Zuercher et al., 2016).

A study carried out in a rat resuscitation model investigated the effect of combined helium pre/postconditioning on brain injury (Aehling et al., 2018). Rats were administered 70% helium for 5 min before CA and 30 min after CPR. Hippocampal CA1 tissues were excised to evaluate neurological degeneration after 2 h, 4 h, or 7 d, and a tape-removal test was adopted to detect cognitive function after 7 d. The results revealed that helium pre/postconditioning tended to decrease apoptosis in the brain, but had little influence on numbers of viable neurons. Results of the tape-removal test revealed no significant differences at 1, 3, or 7 d after CPR (Aehling et al., 2018). However, in an HI model in developing brains of rat pups pretreated with helium, increased numbers of viable neurons were observed in the cortex and hippocampus, and caspase-3 activity and brain infarct size were suppressed (Liu et al., 2011). Compared with the control group, helium-treated animals also did better in Morris water maze and postural reflex tests 5 w after modeling, indicating relatively well-recovered cognitive and sensorimotor functions (Liu et al., 2011). Differences in measurements used to test neurological functions and the timing/duration of helium conditioning inevitably result in different outcomes.

Temperature management may be another contributor to the effect of helium. David et al. found that helium treatment administered at 25°C reduced brain infarction and motor deficits, while administration at 33°C exerted no neuroprotection (David et al., 2009). They also found that helium provided neuroprotection by inducing hypothermia and the extent of body temperature reduction mainly depended on the gas temperature at which helium was applied (David et al., 2009).

The effects of different starting times of helium application on brain recovery were evaluated in a model of thromboembolic brain ischemia (Haelewyn et al., 2016). The results show that helium administered before or together with tissue plasminogen activator (tPA) therapy increased the risk of inhibiting the benefit of tPA-induced thrombolysis; however, administration after tPA-induced reperfusion could yield neuroprotective effects. Brain hemorrhage is the consequence of tPA-mediated adverse proteolytic processes during treatment of brain ischemia. Helium, like xenon, may directly bind within the catalytic domain of tPA to account for its protection when administrated after reperfusion (Haelewyn et al., 2016). Because helium inhalation at 25°C or below produces hypothermia, a condition shown by itself to reduce the thrombolytic and proteolytic properties of tPA (Tang et al., 2013), it is difficult to determine whether binding with the catalytic site or helium-induced hypothermia mediates the neuroprotective effect of helium.

Following administration of helium and xenon at a concentration of 37.5% to brain slices subjected to OGD, David et al. found that the mixture of helium and xenon could inhibit the increase of lactate dehydrogenase induced by OGD (David et al., 2017). Moreover, this mixture reduced the catalytic and thrombolytic efficiency of rat tPA to a similar extent as Xe-50.

Xe-He-37.5 has some new properties compared with xenon alone, such as a lower molecular weight, higher thermal conductivity, and increased specific heat, making it an efficient alternative to Xe-50 (David et al., 2017). These findings indicate that a combination of 37.5% helium and 37.5% xenon could both produce neuroprotection and reduce the adverse side effects of rat tPA. This finding provides an exciting prospect for the treatment of thromboembolic brain insults. Indeed, it may be advantageous to investigate the protective effect of Xe-He-37.5 on other organs.

## 5 Conclusion

The noble gases manifest a wide range of biological properties despite their chemical inertness, including analgesia, anaesthesia, neuroprotection, tissue protection, anti-apoptoticity, cytoprotection, ischemic–perfusion injury prevention, anti-convulsion, and effects on memory. The present review summarizes studies evaluating the pharmacology and clinical uses of several inert gases as neuroprotectants. The findings demonstrate significant promise for these gasses as therapeutic agents. However, the expense of xenon as it is currently used clinically is a significant barrier to its wider use, which would offer potential benefits to patients in terms of reduced tissue damage from hypoxic events and improved outcomes after ischemic events. Promising delivery methods have recently emerged using microbubbles and liposomes, with the option of controlled delivery *via* external stimuli such as ultrasound (Chakkarapani et al., 2009; Peng et al., 2013; Dingley et al., 2015). These delivery systems may also allow for cheaper, more abundant, lower atomic weight noble gases to be used for medical purposes without the need for hyperbaric administration by increasing their solubility in blood and tissues under normal atmospheric pressure. These improvements in delivery efficiency would make noble gases viable candidates for novel therapeutics. Helium reportedly exerts neuroprotective effects when applied in combination with xenon in equal concentration. The mixture of xenon and heilum reduces the use of xenon while maintaining a similar effect to xenon at a higher concentration and inheriting the advantages of helium. Argon, the third most abundant element in our atmosphere, is inexpensive and neuroprotective. Moreover, unlike xenon, argon has no sedative properties and hence is less likely to affect the neurologic status of patients. Argon is easily applied (*via* face mask) and devoid of toxicity, making it possible to extend argon treatment for patients suffering from stroke in the acute phase. Although argon holds promise as a valuable tool for protecting the brain, important parameters such as treatment duration, timing of application, accompanying oxygen concentration, effects of sex and age, and potential temperature effects must be systematically studied and defined before clinical trials can be justified and appropriately designed.
